# Microporous Borocarbonitrides B_x_C_y_N_z_: Synthesis, Characterization, and Promises for CO_2_ Capture

**DOI:** 10.3390/nano13040734

**Published:** 2023-02-15

**Authors:** Rimeh Mighri, Umit B. Demirci, Johan G. Alauzun

**Affiliations:** 1 Institut Charles Gerhardt, Univ Montpellier, CNRS, ENSCM, 34095 Montpellier, France; 2 Institut Europeen des Membranes, IEM–UMR 5635, Univ Montpellier, CNRS, ENSCM, 34095 Montpellier, France

**Keywords:** borocarbonitride, boron nitride, ethylenediamine bisborane, microporosity, CO_2_ capture

## Abstract

Porous borocarbonitrides (denoted BCN) were prepared through pyrolysis of the polymer stemmed from dehydrocoupled ethane 1,2-diamineborane (BH_3_NH_2_CH_2_CH_2_NH_2_BH_3_, EDAB) in the presence of F-127. These materials contain interconnected pores in the nanometer range with a high specific surface area up to 511 m^2^ · g^−1^. Gas adsorption of CO_2_ demonstrated an interesting uptake (3.23 mmol · g^−1^ at 0 °C), a high CO_2_/N_2_ selectivity as well as a significant recyclability after several adsorption–desorption cycles. For comparison’s sake, a synthesized non-porous BCN as well as a commercial BN sample were studied to investigate the role of porosity and carbon doping factors in CO_2_ capture. The present work thus tends to demonstrate that the two-step synthesis of microporous BCN adsorbent materials from EDAB using a bottom-up approach (dehydrocoupling followed by pyrolysis at 1100 °C) is relatively simple and interesting.

## 1. Introduction

Gas purification is among the urgent challenges in environment protection. To this aim, many technologies have been employed for gas capture [1], among which the adsorption process using solid materials seems to be the most favorable due to its simple and efficient implantation, low energy consumption and ease of reusability [2].

In recent decades, many materials have been developed and studied for gas (CO_2_, H_2_, O_2_, etc.) purification or storage [3,4,5,6]. Among all materials considered for these applications, porous materials tend to be the most promising [5]. In terms of porosity (micro-, meso-, and macroporosity), structure (crystalline, amorphous) or composition (oxides [7], hybrids [8], MOFs [9], zeolites [10], ceramics [11], etc.), the variety of porous materials is extremely vast [12]. In order to choose the right material, several features have to be considered for this specific application: they have to combine a high adsorption uptake, an affinity or selectivity toward the target component, have good tolerance for extreme conditions, be recyclable and be available at a relatively low cost. As for gas adsorption, carbon dioxide is often studied. For instance, 9.1 mmol · g^−1^ of CO_2_ uptake was reached [13] with a PCN-124 MOF material with a specific surface area (SSA) of 1372 m^2^ · g^−1^, while 1.3 mmol · g^−1^ was obtained with a zeolite loaded hybrid foams [14]. This latter material has the ability to adsorb CO_2_ selectively 27 times higher than N_2_.

Within the long list of porous materials with potential for gases and dyes adsorption, borocarbonitrides (BCN) with good chemical and thermal stability may be of certain importance. Borocarbonitrides and more precisely hexagonal BCN have been proven interesting for applications in electrocatalysis [15], heat storage applications [16] as well as graphene-related technology [17]. However, carbon-doped boron nitrides and BCN are also of great interest for CO_2_ capture [18,19]. To this end, several BCN have been studied such as BCN aerogel as depicted by Tian et al. [20]. Their material, having a specific surface area of 726 m^2^ · g^−1^ and a pore volume of 0.60 cm^3^ · g^−1^, was able to adsorb 0.95 mmol∙g^−1^ of CO_2_ (at 760 mm Hg), which was seven times higher than the amount adsorbed by BN. In addition, the selectivity of CO_2_ uptake over N_2_ reached 11.3.

Unfortunately, these materials need many steps to combine good composition, structure and porosity. For instance, templating technique using mesoporous graphitic carbon nitride (mpg-C_3_N_4_) as a reactive template was coupled with pyrolysis method to form an ordered mesoporous BCN sample [21]. In another work, a combination of chemical vapor deposition (CVD) and polymer-derived ceramic (PDCs) routes was employed to prepare FAU zeolite templates, which were later infiltrated by carbon. The resulting carbonaceous zeolite replica was mixed with polyborazylene then pyrolyzed to obtain the final BN-based material with a specific area of 570 m^2^ · g^−1^ [22]. Simple and interesting ways exist but with lower control of the atomic composition or long-range structure. Such materials have been usually prepared following different synthetic approaches [23]. Several of them consist of thermal treatment of a mixture of boric acid or sodium borohydrides with a carbon source precursor (e.g., urea, activated charcoal, and glucose) [24,25,26].

The present work tends to synthesize a BCN material following a simple approach and to add some porosity in order to enhance the adsorption properties of CO_2_. Herein, we developed a synthesis protocol by adapting the thermal degradation of ethane 1,2-diamineborane (BH_3_NH_2_CH_2_CH_2_NH_2_BH_3_, EDAB) as depicted by Leardini et al. and Martin et al. [27,28]. EDAB was used as a BCN precursor in order to obtain a final ceramic with a B/C/N ratio of approximately 1/1/1. EDAB is a solid alkyl-containing amine-borane adduct containing an equal amount of each of the desired elements. This molecule has already been studied for hydrogen storage applications, due to its high available hydrogen content (11.4 wt%) as well as the moderate conditions needed for the release of hydrogen [29,30]. Yet, the potential of this amine borane goes well beyond the hydrogen storage application [31].

The main aim of the present investigation is to control the porosity of BCN (e.g., specific surface area) in order to obtain an efficient adsorbent for CO_2_ separation with some ability to reversibility as well as a good selectivity toward CO_2_ over N_2_.

To produce some porosity in the final material, EDAB was dehydrocoupled in the presence of Pluronic F-127 as porosity agent in an organic solvent before pyrolysis. F-127 is a widely used tri-block non-ionic copolymer with a composition of (PPG)_100_(PEG)_65_(PPG)_100_, PPG standing for poly-propylene-glycol and PEG for poly-ethylene-glycol. This compound has been mainly used to synthesize mesoporous silica [32] but can also be used for other porous materials [33]. Very few studies have reported the use of F-127 as a porosity agent for the synthesis of porous BN-based materials. Xiong et al. reported the synthesis of metal-free porous BN using boric acid and melamine along with F-127 as a surfactant, which was compared to P-123 [34]. To the best of our knowledge, F-127 had not yet been used for the synthesis of borocarbonitrides. The synthesized material having the optimum specific surface was then characterized using conventional techniques such as scanning electron microscope (SEM), X-ray powder diffraction (XRD), ^11^B solid-state NMR and Fourier-transform infrared spectroscopy (FTIR). Detailed adsorption experiments were conducted to determine the maximum adsorption uptake and kinetics. The general idea consists of developing materials with physical adsorption capabilities. This usually facilitates an easy gas storage reversibility, as the interactions with the material surface are weak. Within this model, the physical adsorption can be explained by several mechanisms: thermodynamics, kinetics, steric and diffusion [35]. Our experiments can thus be easily compared to many of the materials already developed as gas adsorbents, following this physical adsorption model [36].

## 2. Materials and Methods

### 2.1. Materials Synthesis

The following chemicals were purchased from Merck-Sigma-Aldrich and used without further purification: ethylene diamine EDA (≥99.5%), borane dimethyl sulfide complex BH_3_-SMe_2_, Pluronic F-127, anhydrous diethyl ether DE (≥99%), anhydrous tetrahydrofuran THF (≥99.9%), anhydrous diglyme (99.5%) and boron nitride BN (1 μm, 98%).

The protocol used for the synthesis of EDAB is described hereafter. Typically, 2.3 mL of BH_3_-SMe_2_ in excess (24.2 mmol) was dissolved in 10 mL of DE in an argon-filled Schlenk flask. The mixture was stirred for 1 h at 500 rpm. The flask was placed in an ice filled crystallizer dish to cool down to 0 °C. Then, 0.76 mL of EDA (11.4 mmol) was added dropwise using a syringe pump at a 1 mL · h^−1^ rate. Afterwards, the mixture was allowed to return to room temperature and kept under stirring for 5 h. The mixture was filtrated to separate the solid-state EDAB, and the liquid fraction consisted of the solvent, SMe_2_ and unreacted BH_3_-SMe_2_. The remaining white solid was dried under vacuum at 80 °C for 24 h (yield of 93 %). Due to air and moisture sensitivity of EDAB, it was stored and used in an argon-filled glovebox with H_2_O and O_2_ content of less than 0.1 ppm (MBraun Labstar).

The BCN materials were synthesized as follows, where multiple conditions such as the solvent, the use of a porosity agent at different ratios and the temperature for dehydrocoupling were varied. In a typical procedure, 300 mg of EDAB and 96 mg of F-127 were dissolved in 6 mL of THF using the Schlenk line technique. The experiment will be denoted as 3/1 mass ratio hereafter. The mixture was stirred at 500 rpm for 1 h. The homogenous clear solution was transferred into a 45 mL PTFE lined autoclave and placed in an oven to undergo dehydrocoupling at 120 °C for 72 h under autogenous pressure. After evaporation of the solvent under vacuum at 40 °C for 24 h (80 °C when using diglyme), the resulting borazine-linked polymer was placed in an alumina crucible and pyrolyzed at 1100 °C with a ramp of 10 °C · min^−1^ during 90 min in a tubular furnace under an argon flow of 50 mL.min^−1^. The different synthesis conditions are summarized in Table 1.

### 2.2. Materials Characterization

The materials textural properties were studied by nitrogen adsorption–desorption at −196 °C using 3Flex surface analyzer from Micromeritics. Before the analysis, the samples were degassed at 250 °C for 15 h under a vacuum level of 1.33 10^−3^ mbar. The Brunauer–Emmett–Teller model (BET) was applied for specific surface area (SSA) measuring in the range of a relative pressure (P/P°) between 10^−5^ and 0.1. The total pore volume was calculated based on adsorbed nitrogen volume at a relative pressure of 0.99. Finally, microporous surface area and volume (pore width w ≤ 2 nm) were measured using the t-Plot model in the linear range of P/P° between 0.2 and 0.5. The pore size distribution of micropores was evaluated using non-local density functional theory (NLDFT) calculation method using SAIEUS software. Scanning electron microscopy (SEM) images were obtained using a Hitachi S4800 microscope to study the morphology of the different samples.

The elemental compositions of the materials (carbon, nitrogen and hydrogen) were measured by the Vario Micro cube analyzer (Elementar). The boron contents were determined using an iCAP 7400 Duo Inductively Coupled Plasma-Optical Emission Spectrometer ICP-OES from Thermo Scientific with the following wavelengths: 249.773, 249.677, 208.959, 182.641 and 136.246 nm. Prior to analysis, 25 mg of the material was mineralized by dissolving it in a mixture of hydrochloric and nitric acids, transferred in an autoclave and heated at 180 °C for 16 h. The resulting solution was diluted in water before analysis. Finally, the O content was determined using SEM-EDX (Energy Dispersive X-ray spectroscopy) analyses performed on a Zeiss EVO HD15 electron microscope.

Thermal stability of the materials was studied by thermogravimetric analysis (TGA) under synthetic air using STA 409 PC Luxx thermal analyzer of Netzch. The samples were placed in an alumina crucible and heated up to 1100 °C with a ramp of 5 °C · min^−1^.

For structure analysis, powder X-ray diffraction (XRD) was used. The patterns were recorded on an X’Pert Powder Spinner with a Cu-Kα source (Kα = 0.154 nm).

Raman spectra were recorded using Horiba Jobin Yvon LabRAM Aramis spectroscope with a 473 nm laser. FTIR spectroscopy analyses were performed using PerkinElmer Spectrum two spectrometer with attenuated total reflectance (ATR) mode. A background spectrum was recorded under air before each analysis. ^11^B solid-state nuclear magnetic resonance (NMR) experiments were performed on a Varian VNMR4000 spectrometer with a resonance frequency 128,355 MHz equipped with a 9.39 T wide-bore magnet using a 3.2 mm magic angle spinning (MAS) probe with a spinning rate of 20 kHz.

### 2.3. CO_2_ Adsorption Measurement

3Flex analyzer was employed for CO_2_ uptake measurements using volumetric method. For CO_2_ adsorption isotherms, samples were degassed at 250 °C for 15 h under a vacuum level of 1.33 10^−3^ mbar, then measured at 1 bar and different temperatures. The adsorption uptake at a given temperature is equivalent to the highest adsorbed quantity of the corresponding isotherm located at a pressure of 1 bar.

The affinity between an adsorbate and the surface of an adsorbent is a key factor in adsorption mechanisms. Therefore, the isosteric adsorption enthalpy was calculated using the method of Clausius–Clapeyron (C-C) according to the following Equation (1):

(1) $$\Delta H_{ads}=R{\left[\frac{\partial \ln P}{\partial \;\left(\frac{1}{T}\right)}\right]}_{q}$$

where [∆*H*]*_ads_* is the isosteric adsorption enthalpy (kJ · mol^−1^), *R* is the ideal gas constant (8.314 J · mol^−1^ · K^−1^) and *q* is the adsorbed quantity of CO_2_ at a pressure *P* and temperature *T* [37].

Equation (2), obtained by solving Equation (1), was applied to the obtained isotherms at 0 and 35 °C.

(2) $$\Delta H_{ads}=-\left(\frac{RT_{1}T_{2}}{T_{2}-T_{1}}\ln\frac{P_{2}}{P_{1}}\right)$$

Selectivity of CO_2_ compared to N_2_ was measured according to Ideal Adsorption Solution Theory (IAST) method at atmospheric conditions applied to the flue gas composition (15% CO_2_, 75% N_2_ and 10% other gases) [38]. The equation of the IAST model goes as the following:

(3) SCO2N2=q1q2P1P2

where *q*_1_ is the adsorbed quantity of CO_2_ at *P*_1_*, q*_2_ is the adsorbed quantity of N_2_ at *P*_2_*, P*_1_ = 0.15 bar and *P*_2_ = 0.75 bar.

Another critical criterion for practical CO_2_ adsorption applications is the ease of recyclability of the materials. The reuse and recyclability of the BCN materials was tested at 25 °C and 1 bar. In a typical procedure, a CO_2_ adsorption experiment is performed as described earlier and considered as the first cycle uptake. The sample was then degassed under vacuum at 150 °C for 2 h and tested again at the same temperature for the next cycle. The recyclability percentage is calculated after each cycle as follows (and taking a 5th cycle as example):

(4) $$Recyclability\;\left(\%\right)=\frac{Qads_{Cycle\;5}}{Qads_{Cycle\;1}}\times 100$$

## 3. Results

### 3.1. Characterization of BCN

The textural properties of all the studied materials are summarized in Table 1. The corresponding nitrogen adsorption–desorption isotherms are presented in Figure S1. The sample BCN-1, synthesized without using a solvent, showed very low porosity. Therefore, experimental conditions were varied to obtain porous materials. First, the use of a solvent was studied. Solvothermal dehydrocoupling resulted in an increase in the SSA, with 57 m^2^ · g^−1^ when using diglyme and 134 m^2^ · g^−1^ when using THF. According to IUPAC classification [39], the former sample, BCN-2, presents a type II isotherm with a H3 hysteresis loop associated with macroporous materials. The latter sample, BCN-3, presents a type I isotherm, typical of microporous materials having relatively small external surfaces along with a H4 hysteresis loop indicating the existence of narrow slit-like pores. The use of F-127 as a porosity agent was also investigated at different mass ratios. As represented in Table 1 and Figure S1, varying the EDAB/F-127 ratio has an effect on the porosity. In the case of BCN-4, we mainly obtained a combination of type I and type IV isotherms, suggesting that it presents both micro- and meso-porous features with a SSA of 190 m^2^ · g^−1^ and a microporous volume of 19%. Upon decreasing the F-127 content, microporous materials were obtained, as is the case of for BCN-5, BCN-6 and BCN-7, with SSA in the range of 433 to 506 m^2^ · g^−1^ and a pore volume higher than 50%. This difference in porosity might be related to the size of the self-assembled F-127 micelles. The higher F-127 content in BCN-4 might have resulted in a higher number of F-127 micelles, which would aggregate during the dehydrocoupling step and leave wider pores in the final ceramic after the pyrolysis treatment. A similar behavior has been observed by Dunophy et al. [40] during the synthesis of mesoporous silica using Pluronic F-127. They reported an increase in the average pore size from 8 to 16 nm upon increasing the F-127/SiO_2_ molar ratio due to the F-127 micelles aggregation. Finally, dehydrocoupling temperature seems to affect the materials porosity where a higher SSA of 506 m^2^ · g^−1^ was obtained when heating at 120 °C compared to 433 m^2^ · g^−1^ at 90 °C with the same EDAB/F-127 ratio. However, heating at higher temperatures, at 150 °C and 180 °C in the case of BCN-9 and BCN-10, respectively, was found to be detrimental to porosity. This is likely due to the boiling of F-127 (b.p. 149 °C) during dehydrocoupling. Among all the synthesized samples, we were mostly interested in the highest SSA material with the highest microporous volume, as it is usually considered as the most promising features for carbon dioxide capture [18].

**Figure 1:**
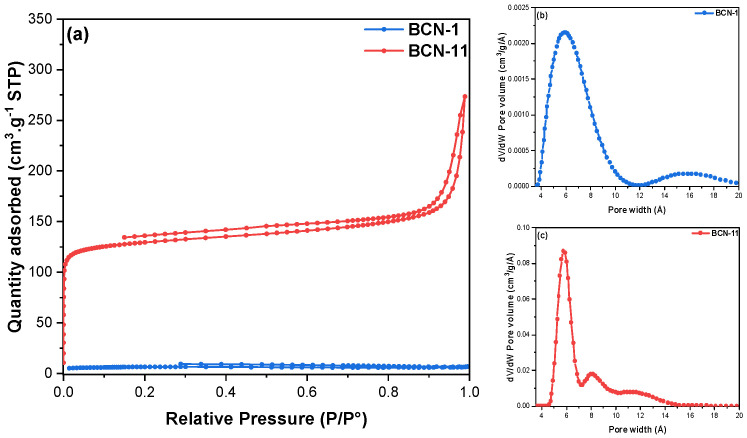
(**a**) Nitrogen adsorption–desorption isotherms at −196 °C of BCN−1 and BCN−11; (**b**,**c**) Pore size distribution of BCN−1 and BCN−11 by NLDFT method.

BCN-11 presents the highest SSA of 511 m^2^ · g^−1^ and pore volume of 0.35 cm^3^ · g^−1^ (60% of microporous volume). Its isotherm (Figure 1a) shows a type I isotherm with a H4 hysteresis loop characteristic of a microporous volume with narrow slit-like pores (60% of microporous volume). Therefore, it was selected for further characterizations. For comparison purposes, especially the study of porosity impact in adsorption applications, BCN-1 was also closely studied. The nitrogen adsorption–desorption isotherms are depicted in Figure 1. The type of microporosity of these materials was detailed by the study of pore size distribution using NLDFT theory. As shown in Figure 1b,c, BCN-11 presents mainly three peaks centered at around 0.57, 0.80 and 1.2 nm with the higher peak intensity at around 0.57 nm, demonstrating the high density of ultra-micropores (w ≤ 0.7 nm).

**Figure 2:**
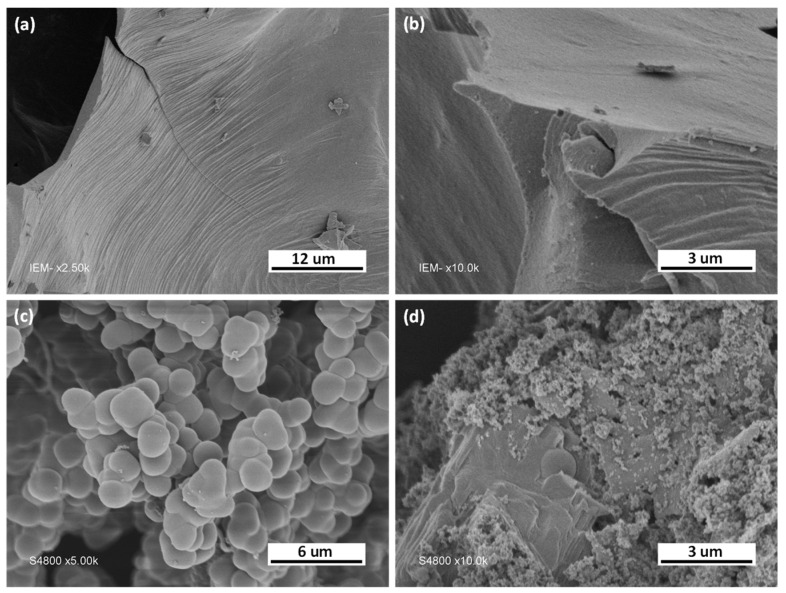
SEM images: (**a**,**b**) BCN−1; (**c**,**d**) BCN−11.

The particles’ morphologies were analyzed by SEM. The SEM images of the BCN materials are shown in Figure 2. They clearly show a dense surface for the non-porous bulk BCN-1. However, the porous BCN-11 features a homogenous distribution of spherical grain-like shape. These spheres have two average diameters of ca. 0.08 µm and ca. 1.8 µm.

Elemental analysis for C, N and H content was performed on all the synthesized samples (Table S1). Carbon content was found to be between 9.4 and 23.8 wt%. The BCN−11, in particular, shows high carbon content, with 23.3 wt%. This, along with the highest SSA, makes it the most suitable candidate for the intended applications (as shown hereafter).

The chemical compositions of BCN-1 and BCN-11 are detailed in Table 2 and can be given as B_1.00_C_0.90_N_1.03_H_1.18_O_0.51_ and B_1.00_C_0.86_N_0.98_H_1.72_O_0.61_, respectively. It is reasonable to conclude that BCN-1 and BCN-11 have comparable chemical compositions. It is worth mentioning that the initial B/C/N ratio of the EDAB molecule is equal to 1/1/1. It has been proven that the reduced ratio of carbon in the pyrolyzed samples, as compared to B and N, is the result of C_x_H_y_ and CO_x_ evolution species during high-temperature pyrolysis of EDAB [27]. This is in good agreement with the atomic ratios obtained. The O content could be explained by the fact that, although this work was performed under inert conditions, small amounts of oxygen could still be present probably due to equipment sealing defaults and small water content even in anhydrous solvents.

**Figure 3:**
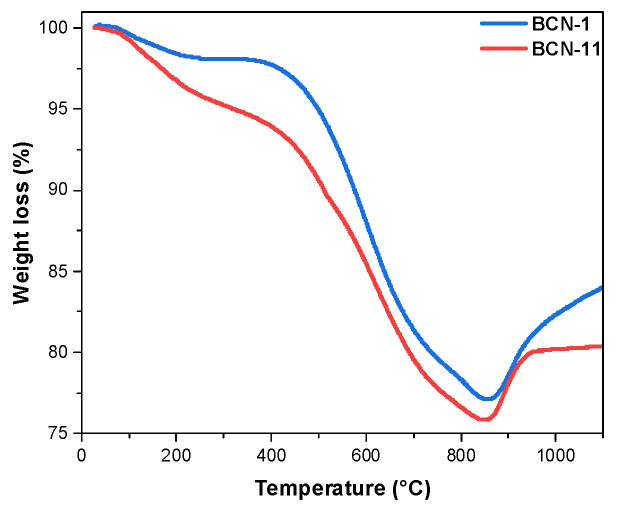
TGA curves of BCN-1 and BCN-11.

According to TGA (Figure 3), BCN-1 and BCN-11 gradually lose mass upon increasing the temperature until 850 °C, owing to their decomposition related to carbon oxidation [21,41] as well as the partial oxidation of nitrogen resulting in the release of nitrogen oxide [42]. On the other hand, the mass increase after 860 °C is related to the oxidation of the unstable boron moieties forming boron oxide [21,43]. As a note of comparison, it has been proven that pure carbon materials, e.g., carbon fibers, lose their total mass at 740 °C while losing only 20% at the same conditions after BN doping [44]. This demonstrates that the BCN materials present higher stability under air than carbon materials.

**Figure 4:**
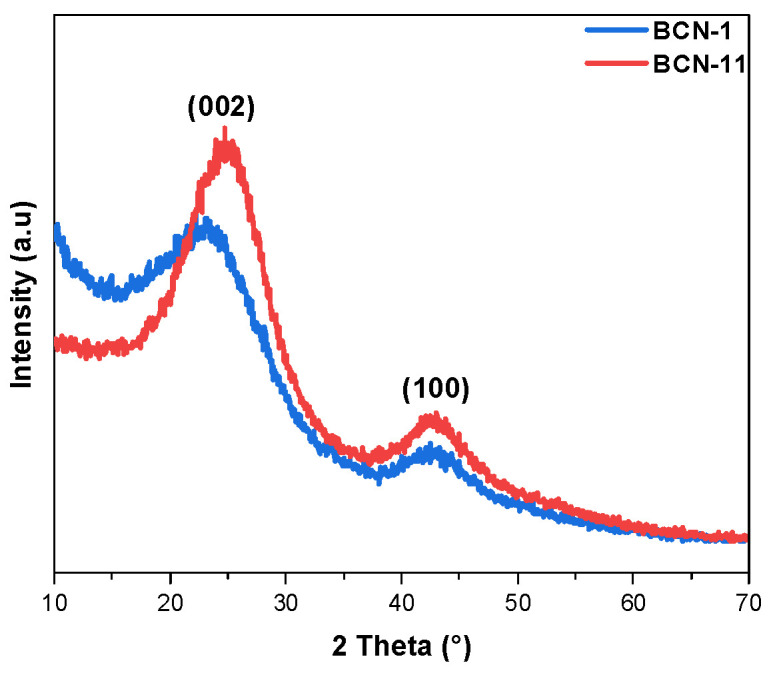
XRD patterns of BCN-1 and BCN-11.

X-ray diffraction patterns of BCN-1 and BCN-11 (Figure 4) show two broad diffraction peaks. The first one is centered at around 24° and the second at around 43°. These peaks positions are characteristics of both hexagonal BCN and hexagonal BN despite the limited crystallinity of the samples. The two peaks correspond to (002) and (100) reflection planes, respectively. In comparison with h-BN, the BCN samples show a general broadening of diffraction planes as well as a shift in the (002) plane towards lower 2θ values (centered at 26.3° for BCN-1 and 24.9° for BCN-11) indicating that the doping of carbon produces structural defects and an increase in interlayer spacing [24,45,46]. The 100 diffraction peaks of all synthesized BCN remain unchanged and are centered at 42.4°. Similarly to BN and BCN materials, graphite shows a diffraction peak at around 26° corresponding to the (002) reflection plane (which is rather sharp). However, it also presents a second peak around 55° assigned to the (004) reflection plane [47]. This implies that C atoms form hybrid BCN domains instead of aggregating to graphite domains.

**Figure 5:**
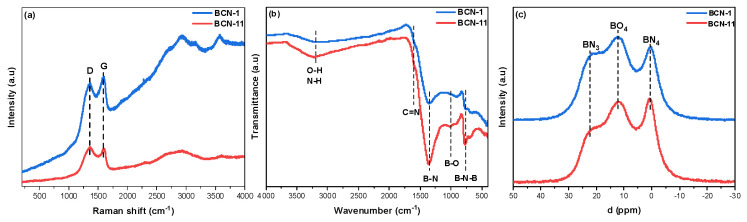
(**a**) Raman spectra of BCN−1 and BCN−11; (**b**) FTIR spectra of BCN−1 and BCN−11; (**c**) ^11^B MAS NMR spectra of BCN−1 and BCN−11.

Figure 5a shows the Raman spectra of BCN-1 and BCN-11. Two strong peaks at 1360 cm^−1^ and 1600 cm^−1^ can be observed. They are assigned to the D and G bands, respectively. The D band results from the lattice distortion of the in-plane substitution heteroatoms, vacancies, or particles edges in the sp^2^ BCN domains. The G band, on the other hand, reflects the structural intensity of the sp^2^ bonds stretching [48,49]. In addition, other peaks at higher shifts were detected, which can be related to higher-order Raman signal of BCN samples (such as the 2D band around 2750 cm^−1^) and can be assigned to the combination of the D and G bands [50]. These results suggest that a BCN is obtained instead of both BN and graphite. Indeed, graphene and graphite do not show (or show very weak) D band along with a sharp 2D band at around 2700 cm^−1^, whereas BN materials show no 2D band [51]. Intensity ratio between the D band and G bands, ID and IG, respectively, are estimated to be 0.94 for BCN-1 and 1.03 for BCN-11, thereby indicating a slightly lower graphitic degree and higher defect density in BCN-11 sample [49]. It is worth noting that Raman spectra of all other synthesized BCN materials (BCN-2 to BCN-10) were similar to BCN-11, along with the same intensity ratio between the D band and G bands (~1.02).

The FTIR spectra (Figure 5b) obtained from BCN-1 and BCN-11 show two bands centered at around 1367 cm^−1^ and 780 cm^−1^ that correspond to the in-plane stretching vibration of *B–N* and to the *B–N–B* bending vibration, respectively. In addition, the broad band at around 3240 cm^−1^ can be attributed to the *O–H* and/or *N–H* bonds. Finally, the two small peaks detected at around 1593 cm^−1^ and 1008 cm^−1^ could be attributed to *C=N* and *B–O*, respectively [52,53]. On the other hand, *C–N* and *B–C* bands, usually located between 1100 cm^−1^ and 1300 cm^−1^ have not been detected, probably due to overlap with the *B–N* band [24,52,54,55]. Solid-state ^11^B MAS NMR spectroscopy was undertaken to study the chemical environment of the B atoms. The spectra in Figure 5c show three distinct signals. In order to identify these signals, we have referred to several studies found in the literature (Table S2). Accordingly, the first signal at around 22 ppm is assigned to the *B–N* bond related to planar BN_3_ groups. The peak at around 12 ppm can be attributed to BO_3_ groups. As for the peak at around 0 ppm, several suggestions have been reported. It is often assigned to tetragonal BN_4_ groups but can also be attributed either to the *B–C* structure or to tetragonal BO_4_. In our case, as the *B–C* bond could not be detected in FTIR analyses, it is reasonable to attribute it to tetragonal BN_4_ groups.

### 3.2. CO_2_ Adsorption Measurements

**Figure 6:**
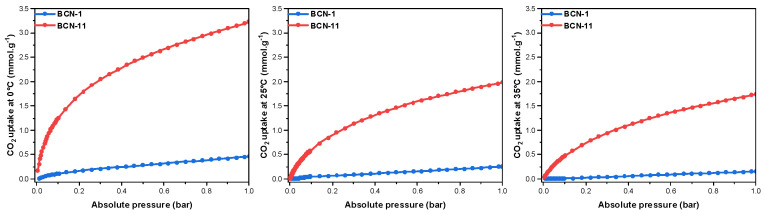
CO_2_ uptakes of BCN samples at 0 °C, 25 °C and 35 °C.

CO_2_ adsorption isotherms of BCN-1 and BCN-11 (Figure 6) were measured at different temperatures and an absolute pressure up to 1 bar. To investigate the effect of carbon doping, a commercial non-porous (or macroporous) BN showing a SSA of 13 m^2^ · g^−1^ (Figure S2), was also analyzed. The CO_2_ uptakes of all the studied samples are shown in Figure 6 and Figure S3, and the main results are summarized in Table 3. BCN-11 shows much higher CO_2_ uptake (3.23 mmol · g^−1^ or 142 mg · g^−1^, at 0 °C) compared to BCN-1 (0.46 mmol · g^−1^ or 20 mg · g^−1^, at 0 °C) which proves the effect of porosity on the adsorption uptake. This could further be accounted for the presence of ultra-micropores in the BCN-11, formerly proven to enhance CO_2_ adsorption due to compatibility of ultra-micropores size (<0.7 nm) with CO_2_ kinetic diameter [18]. BCN-1 has better adsorption uptake than commercial BN (0.09 mmol · g^−1^ at 0 °C), which confirms that carbon doping is also an enhancing factor for CO_2_ uptake.

This can probably be explained by the formed structural defects and the tuning of surface charges, which enhances interactions between acidic CO_2_ and basic sites such as N groups. In fact, with structural defects, mostly boron vacancies are obtained, which limit the repulsion interactions with CO_2_ while the lone pair electrons of nitrogen can form electrostatic interactions with the acidic adsorbate [18,19].

**Figure 7:**
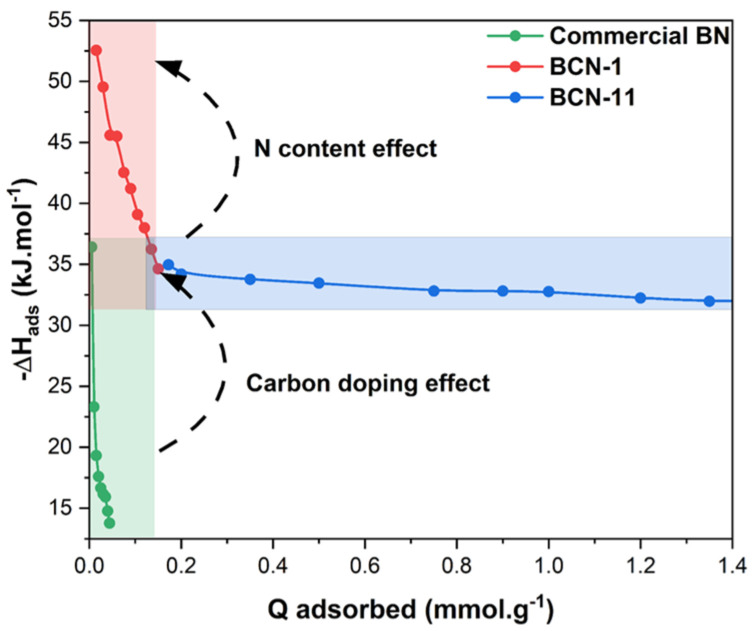
Isosteric adsorption enthalpies of Commercial BN, BCN−1 and BCN−11 as a function of CO_2_ adsorbed quantity.

To have a better understanding of the CO_2_ adsorption phenomenon, the isosteric adsorption enthalpy ΔH_ads_ was calculated to evaluate the interaction strength between CO_2_ and the surface of the adsorbents. As shown in Figure 7, the adsorption enthalpies obtained are in the range of −34 to −53 kJ · mol^−1^ at CO_2_ uptakes between 0.015 and 0.15 mmol · g^−1^ for BCN-1. Those of BCN-11 varied from −31 to −35 kJ · mol^−1^ at CO_2_ uptakes between 0.02 and 1.74 mmol · g^−1^. The latter values fit well in the range of physisorption (>−50 kJ · mol^−1^) and are lower than those of chemisorption (<−60 kJ · mol^−1^), which indicates that CO_2_ is mainly physisorbed onto the BCN-11 surface. Meanwhile, the former enthalpy values rather approach the limits of chemical adsorption [56]. Commercial BN presented much higher adsorption enthalpies (which means lower isosteric heat of adsorption) between −14 and −36 kJ · mol^−1^ indicating weaker interactions with CO_2_. This is in good agreement with reported studies on element doping, such as carbon. For example, Chan et al. [18] obtained higher isosteric heat of adsorption by doping hexagonal BN with carbon; it increased from 27–28 kJ · mol^−1^ to 32–35 kJ · mol^−1^, thus improving the CO_2_ uptake performance (1.16–1.66 mmol · g^−1^ for BN samples and 3.74–3.91 mmol · g^−1^ for BCN samples at 25 °C, 1 bar). In the same context, Tian et al. [20] reported a CO_2_ adsorption uptake of BN of 0.13 mmol · g^−1^ (25 °C, 1 bar) which increased to 0.95 mmol · g^−1^ (25 °C, 1 bar). This was accompanied, according to DFT calculations, with an increase in the adsorption heat, going from 6.16 kJ · mol^−1^ for BN to 7.10 kJ · mol^−1^ for BCN and even higher for NH_2_-BCN with an adsorption heat of 7.26 kJ · mol^−1^.

Moreover, the adsorption enthalpy seems to be higher at low CO_2_ uptakes and tends to decrease with an increase in the surface coverage. This highlights the heterogeneity of the materials surface and enhancement of diffusion by adsorption of CO_2_ in narrow pores [57].

**Figure 8:**
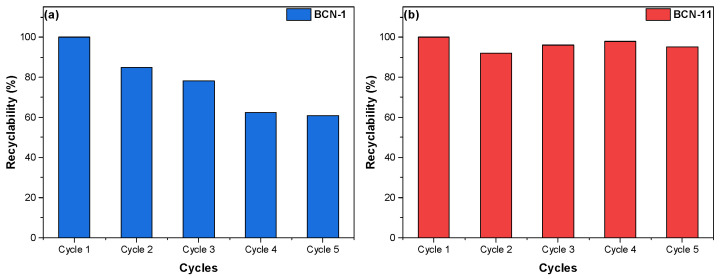
CO_2_ recyclability at 25 °C of (**a**) BCN-1 and (**b**) BCN-11.

Figure 8 shows the CO_2_ uptake of BCN-1 and BCN-11 after five adsorption–desorption cycles. As one can see, BCN-11 shows a good recyclability with a recycling percentage of 96%. However, the uptake of BCN-1 decreased to 60% at the 5th cycle. These results are in good agreement with the aforementioned isosteric enthalpy values. The ease of recyclability with BCN-11 is related to the dominant physical nature of adsorbent–adsorbate interactions. With respect to BCN-1, it presents possible chemical interactions with CO_2_, probably due to its slightly higher N content, which may explain why its recyclability is partially compromised. It is known that, in addition to microporosity, a higher density of N groups helps increase the isosteric heat of adsorption [58,59].

**Figure 9:**
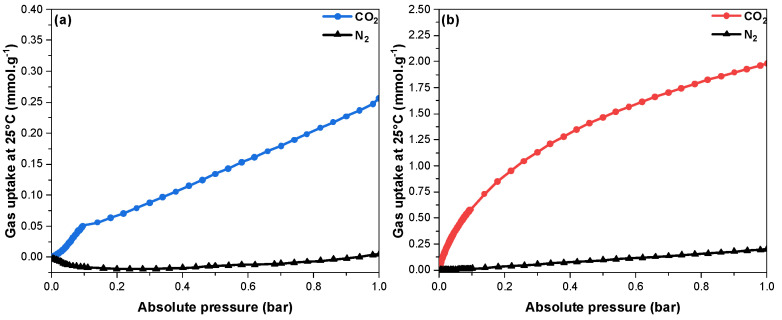
CO_2_ and N_2_ adsorption isotherms at 25 °C of (**a**) BCN−1 and (**b**) BCN−11.

In order to capture CO_2_, from either air or flue gas, the selectivity for CO_2_ adsorption as opposed to N_2_ represents a key factor for these targeted applications. Figure 9 shows the adsorption isotherms of both N_2_ and CO_2_ at 25 °C. One can see that the amount of CO_2_ adsorbed was much higher than that of N_2_ in any pressure range. This was further confirmed by the calculation of the selectivity value based on the IAST method for flue gas composition evaluated to be equal to 26 for BCN-11. However, since the N_2_ adsorption for BCN-1 at 25 °C is too low, it leads the selectivity equation to diverge to infinity based on a null value of *q_2_*, which is considered insignificant. This falls under the category of the theory’s limitations, when working at low pressure [60].

Many types of porous adsorbents have been studied for CO_2_ adsorption. Wang et al. [61] published a well-detailed and exhaustive review paper reporting CO_2_ capture performances of different solid adsorbents. Some examples are presented in Table S3. For instance, reported carbon-based materials (having high surface areas) had CO_2_ uptakes between 0.5 mmol · g^−1^ for CuO loaded porous carbon (at 25 °C) [62] and 8.9 mmol · g^−1^ for activated carbon spheres (at 0 °C) [63] but relatively low selectivity [61,64]. For zeolites and silica-based materials, the strong interaction with the surface led to chemisorption of CO_2_, which consumes energy and thus disrupts their recyclability. MOFs are other candidates for CO_2_ capture owing to very high surface areas (>1000 m^2^ · g^−1^) and controllable pore size. However, they are sensitive to moisture, and their synthesis procedure could be complicated, thus leading to a high production cost.

On the other hand, BN-based materials present not only high surface areas but also great chemical inertness, oxidation resistance and thermal stability. Additionally, carbon doping strengthens electronic interactions and introduces structural defects to BN structure. Table 4 shows a comparison of the adsorption uptakes of some BN-based adsorbents reported elsewhere, as well as their CO_2_/N_2_ selectivity at 1 bar. Those materials present different textural properties and chemical compositions, which has led to distinct adsorption performances. Tian et al. [20] were able to enhance CO_2_ adsorption uptake of BN by carbon doping with an increase from 0.13 mmol · g^−1^ to 0.95 mmol · g^−1^ (25 °C, 1 bar). Chan et al. [18] obtained much higher uptakes by carbon doping hexagonal BN reaching 3.91 mmol · g^−1^ (25 °C, 1 bar). Liang et al. [21] demonstrated that metal doping by Cu also improves adsorption uptakes. The CO_2_ uptake reached 2.77 mmol · g^−1^ (0 °C, 1 bar). However, the selectivity towards CO_2_ over N_2_ decreased.

As we can observe, our synthesized BCN-11 material (this work) presents good CO_2_ adsorption uptake (up to 3.23 mmol · g^−1^ at 0 °C), comparable to C-doped BN_1100 [58]. It also has great CO_2_/N_2_ selectivity (26), equivalent to the BN650 [38] along with ease of recyclability.

## 4. Conclusions

A series of BCN materials have been synthesized from ethane 1,2 diamineborane (BH_3_NH_2_CH_2_CH_2_NH_2_BH_3_, EDAB) using F-127 as porosity agent. The synthesis integrates two steps, the first one being the dehydrocoupling of EDAB and the second one consisting of pyrolysis of the resulting polymer. The BCN materials are mainly microporous, having specific surface areas up to 510 m^2^.g^−^^1^ and a pore volume of 0.35 cm^3^.g^−^^1^. As demonstrated by N_2_ and CO_2_ adsorptions, the porosity is easily accessible; the pores of the synthesized borocarbonitrides have potential for CO_2_ capture. Indeed, we measured a CO_2_ uptake of up to 3.23 mmol.g^−^^1^ at 0 °C as well as a significant CO_2_/N_2_ selectivity value of 26 according to IAST method. We thus believe that our porous BCN materials are potential alternative to zeolites. Further works are needed to improve the textural properties and thus improve the adsorption capacities.

## Figures and Tables

**Table 1:** The different synthesis conditions of BCN samples.

Sample	Solvent	Dehydrocoupling T°	EDAB/ F-127 Mass Ratio	BET SSA (m^2^ · g^−1^)	Total Pore Volume (cm^3^ · g^−1^)	t-Plot Microporous SSA (%)	t-Plot Microporous Volume (%)
BCN−1	Solvent free	90 °C	F-127 free	23	0.01	97	79
BCN−2	Diglyme	90 °C	F-127 free	57	0.02	-	-
BCN−3	THF	90 °C	F-127 free	134	0.09	96	51
BCN−4	THF	90 °C	2/1	190	0.26	70	19
BCN−5	THF	90 °C	3/1	506	0.30	97	54
BCN−6	THF	90 °C	4/1	433	0.29	95	52
BCN−7	THF	90 °C	8/1	442	0.24	95	62
BCN−8	THF	120 °C	4/1	459	0.24	97	67
BCN−9	THF	150 °C	4/1	19	0.03	16	-
BCN−10	THF	180 °C	4/1	13	0.01	-	-
BCN−11	THF	120 °C	3/1	511	0.35	97	60

(-): insignificant value.

**Table 2:** Chemical composition and atomic ratios of BCN-1 and BCN-11.

Sample	B wt%	C wt%	N wt%	H wt%	O wt%	Atomic Ratios
BCN-1	23.8	23.8	31.65	2.6	18.1	B_1.00_C_0.90_N_1.03_H_1.18_O_0.51_
BCN-11	23.3	22.3	29.6	3.7	21.0	B_1.00_C_0.86_N_0.98_H_1.72_O_0.61_

**Table 3:** CO_2_ uptake performances of the studied materials at 1 bar.

Sample	CO_2_ Uptake in mmol · g^−1^ (in mg · g^−1^)			CO_2_/N_2_ IAST Selectivity	Recyclability after 5 Cycles
	0 °C	25 °C	35 °C		
BCN-1	0.46 (20)	0.26 (11)	0.15 (7)	-	60%
BCN-11	3.23 (142)	1.98 (87)	1.74 (77)	26	96%
BN	0.09 (4)	0.04 (2)	-	-	-

**Table 4:** Comparison of CO_2_ uptake and selectivity of several BN-based porous adsorbents.

Material	SSA (m^2^ · g^−1^)	T (°C)	CO_2_ Uptake (mmol · g^−1^)	IAST CO_2_/N_2_ Selectivity	Reference
BCN-1	23	0	0.46	-	This work
		25	0.26		
BCN-11	511	0	3.23	26	
		25	1.98		
BN	881–1132	25	1.16–1.66	18	[18]
BCN	727–877		3.74–3.91	74	
BN650	235	25	0.47	26.3	[38]
BCN-50	160	25	0.62	-	[54]
BN aerogel	1558	25	0.13	-	[20]
BCN aerogel	726		0.95	11.3	
C-doped BN_1100	387	0	3.71	-	[58]
BNNF	715	0	1.34	18.23	[65]
Cu@BNNF	653		2.77	15.36	

## Data Availability

Not applicable.

## Supplementary Materials

The following supporting information can be downloaded at: https://www.mdpi.com/article/10.3390/nano13040734/s1, Figure S1: Nitrogen adsorption–desorption isotherms at −196 °C of the synthesized BCN materials; Figure S2: N_2_ adsorption–desorption isotherm of commercial BN at −196 °C. The N_2_ sorption isotherm is of type III and is characteristic of a non-porous or macroporous material; Figure S3: CO_2_ adsorption isotherms of commercial BN at 0 and 25 C, 1 bar; Table S1: Elemental analysis of the synthesized BCN materials; Table S2: ^11^B solid-state NMR signals and bond type reported in the literature [66,67,68,69,70,71,72]. Table S3: CO_2_ uptake of different adsorbents according to the literature [73,74,75,76,77].
