# Supplementary material for: Microporous Borocarbonitrides BxCyNz: Synthesis, Characterization, and Promises for CO2 Capture
Source: Nanomaterials (Basel). 2023 Feb 15;13(4):734. doi: 10.3390/nano13040734 (PMC9960740; doi:10.3390/nano13040734)
Supplement: Supplementary file 1 [file nanomaterials-13-00734-s001.zip › nanomaterials-2201165-supplementary.pdf]

Supplementary information

Microporous Borocarbonitrides BxCyNz: Synthesis, Characterization, and Promises for CO2 Capture

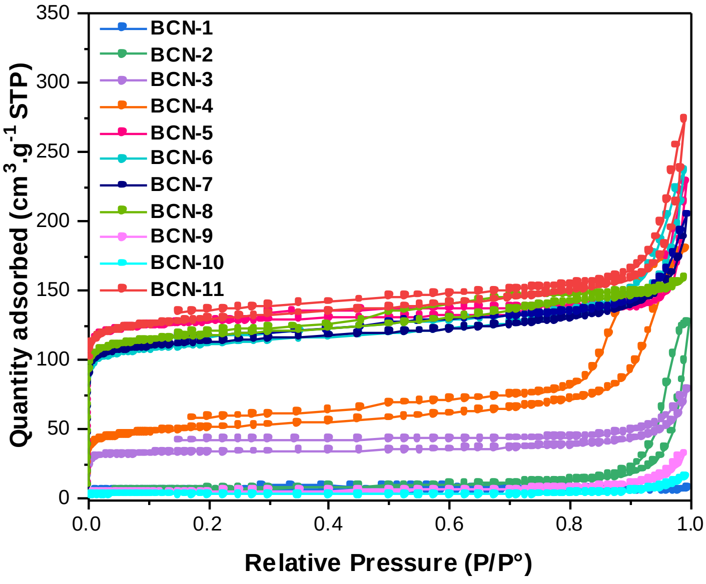

Figure S1: Nitrogen adsorption-desorption isotherms at -196 °C of the synthesized BCN materials.

Table S1: Elemental analysis of the synthesized BCN materials.

Sample	C wt%	N wt%	H wt%
**BCN-1**	23.8	31.65	2.6
**BCN-2**	17.3	26.9	2.5
**BCN-3**	20.1	27.0	2.9
**BCN-4**	15.7	17.5	2.0
**BCN-5**	20.1	26.8	2.8
**BCN-6**	20.3	28.3	3.0
**BCN-7**	17.6	26.4	2.4
**BCN-8**	19.9	27.2	2.9
**BCN-9**	12.5	18.1	1.9
**BCN-10**	9.4	11.6	1.7
**BCN-11**	22.3	29.6	3.7

Table S2: ^{11}B solid-state NMR signals and bond type reported in literature.

**Chemical shift (ppm)**	**Bond type**	**Reference**
0 - 1.25	Tetragonal BO_4	[65]
		[66]
		[18]
	B-C	[67]
	BN_4	[68]
		[69]
11-20.5	Trigonal BO_3	[65]
		[66]
		[67]
20-30	Planar BN_3	[65]
		[70]
		[71]
		[67]
		[68]
30-35	BCN_2	[69]
50	B-C	[71]
	BC_2N	[69]

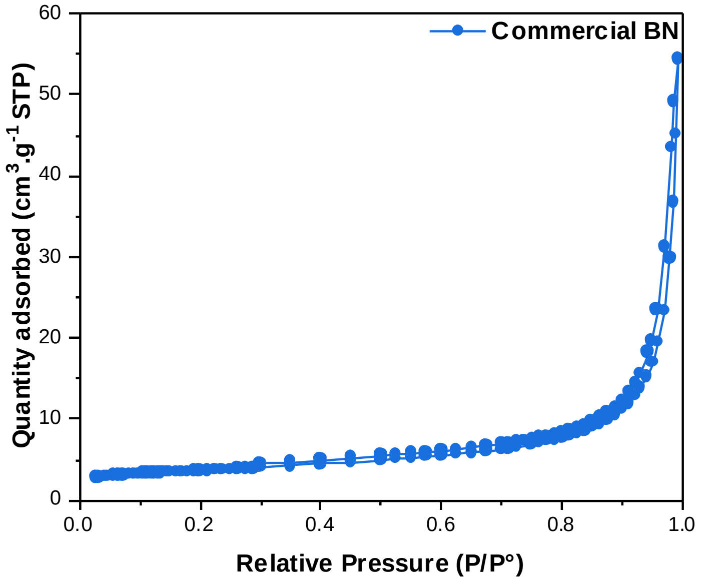

Figure S2: N_2 adsorption-desorption isotherm of commercial BN at -196 °C. The N_2 sorption isotherm is of type III and is characteristic of a non-porous or macroporous material.

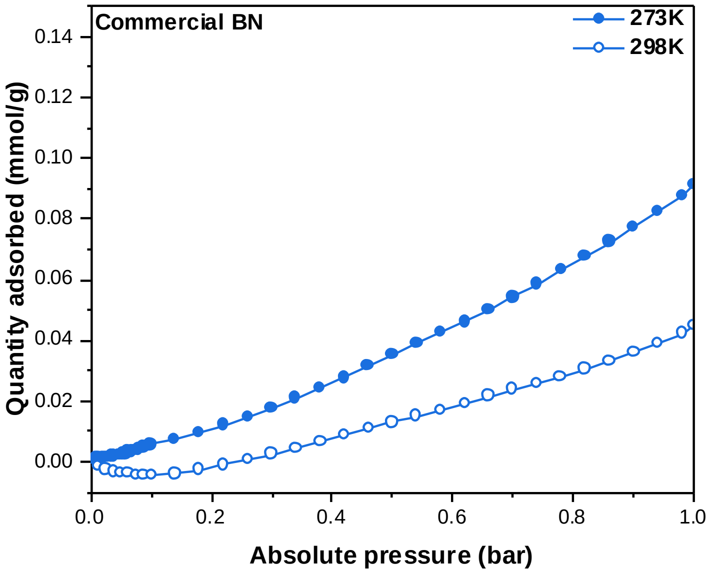

Figure S3: CO_2 adsorption isotherms of commercial BN at 0 and 25 °C, 1 bar.

Table S3: CO_2 uptake of different adsorbents according to literature.

Material	SSA ($m^2.g^{-1}$)	T (°C)	CO_2 uptake ($mmol.g^{-1}$)	IAST CO_2/N_2 Selectivity	Reference
**Carbon-based sorbents**					
**CuO loaded porous carbon**	1510	25	0.5	-	[62]
**N-doped porous carbon**	1700	0	6.2	5.3	[72]
**Activated carbon spheres**	2400	0	8.9	-	[63]
**Zeolite-based sorbents**					
**Zeolite loaded hybrid foams**	400	35	1.3	27	[14]
**Zeolite Ca**	302	25	1.6	-	[73]
**H-SSZ-13**	746	25	4.0	-	[74]
**Silica-based sorbents**					
**APS grafted silica**	200	27	0.7	-	[75]
**Tertiary amine modified silica nanotubes**	348	25	2.3	-	[2]
**APTMS and poly-L-alanine-co-grafted silica**	56-517	25	1.1-3.4	-	[76]
**MOF-based sorbents**					
**2D-MOF**	340	0	2.9	-	[77]
**PCN-124**	1372	0	9.1	-	[13]
